# Inhibition of Ceramide Metabolism Sensitizes Human Leukemia Cells to Inhibition of BCL2-Like Proteins

**DOI:** 10.1371/journal.pone.0054525

**Published:** 2013-01-16

**Authors:** Lavona Casson, Lauren Howell, Lesley A. Mathews, Marc Ferrer, Noel Southall, Rajarshi Guha, Jonathan M. Keller, Craig Thomas, Leah J. Siskind, Levi J. Beverly

**Affiliations:** 1 Division of Hematology and Oncology, Department of Medicine, James Graham Brown Cancer Center, University of Louisville, Louisville, Kentucky, United States of America; 2 Department of Drug Discovery and Biomedical Sciences, South Carolina College of Pharmacy at the Medical School of South Carolina, Charleston, South Carolina, United States of America; 3 Division of Preclinical Innovation, National Center for Advancing Translational Sciences, National Institutes of Health, Bethesda, Maryland, United States of America; 4 Ralph H. Johnson Veterans Affairs Medical Center, Charleston, South Carolina, United States of America; Cincinnati Children’s Hospital Medical Center, United States of America

## Abstract

The identification of novel combinations of effective cancer drugs is required for the successful treatment of cancer patients for a number of reasons. First, many “cancer specific” therapeutics display detrimental patient side-effects and second, there are almost no examples of single agent therapeutics that lead to cures. One strategy to decrease both the effective dose of individual drugs and the potential for therapeutic resistance is to combine drugs that regulate independent pathways that converge on cell death. BCL2-like family members are key proteins that regulate apoptosis. We conducted a screen to identify drugs that could be combined with an inhibitor of anti-apoptotic BCL2-like proteins, ABT-263, to kill human leukemia cells lines. We found that the combination of D,L-threo-1-phenyl-2-decanoylamino-3-morpholino-1-propanol (PDMP) hydrochloride, an inhibitor of glucosylceramide synthase, potently synergized with ABT-263 in the killing of multiple human leukemia cell lines. Treatment of cells with PDMP and ABT-263 led to dramatic elevation of two pro-apoptotic sphingolipids, namely ceramide and sphingosine. Furthermore, treatment of cells with the sphingosine kinase inhibitor, SKi-II, also dramatically synergized with ABT-263 to kill leukemia cells and similarly increased ceramides and sphingosine. Data suggest that synergism with ABT-263 requires accumulation of ceramides and sphingosine, as AMP-deoxynojirimycin, (an inhibitor of the glycosphingolipid pathway) did not elevate ceramides or sphingosine and importantly did not sensitize cells to ABT-263 treatment. Taken together, our data suggest that combining inhibitors of anti-apoptotic BCL2-like proteins with drugs that alter the balance of bioactive sphingolipids will be a powerful combination for the treatment of human cancers.

## Introduction

Cancer cells are a distorted version of their normal counterparts [1]. One of the important distortions that separate cancer cells from healthy cells is the inability to undergo programmed cell death, or apoptosis, triggered by homeostatic processes. However, a peculiar observation is that most cancer cells are capable of undergoing apoptosis following treatment with cytotoxic stimuli. The challenge lies in identifying the particular stimuli that will effectively kill a given cancer, while sparing the healthy cells of the patient’s body. Historically, high doses of toxic compounds have been used, and are still used, to eradicate cancers; however, the unwarranted consequences of this type of regimen are the detrimental side effects that patients experience due to the death of normal cells of the body. By understanding the biochemical and molecular requirements of cancer cells, it may be possible to avoid these detrimental side effects by combining lower doses of drugs that trigger apoptosis preferentially in cancer cells. Two families of molecules have been actively studied that may fulfill these criteria and give therapeutic benefit to cancer patients. The first is the BCL2-family of proteins and the second are bioactive sphingolipids. Both families can be sub-divided into members that possess the ability to either cause or prevent apoptosis and modulators of these molecules are being explored as potential cancer therapeutics [2], [3], [4], [5].

Anti-apoptotic BCL2-like proteins regulate critical aspects of apoptosis by inhibiting mitochondria outer membranes permeabilization (MOMP), a requisite step in the initiation of the apoptotic pathway that results in the release of proteins from the mitochondrial intermembrane space to the cytosol where they activate caspases and DNases necessary for the execution of the cell [3], [6], [7]. It has been demonstrated that many cancer cells are critically dependent on the activity of anti-apoptotic BCL2-like proteins to maintain survival [8], [9], [10]. As such, multiple inhibitors of BCL2-like proteins are currently being explored in clinical trials as potential therapeutics. One such compound is ABT-263 (or first generation compound, ABT-737), is a small molecule designed to interact with three of the BCL2-like proteins, BCL2, BCLx_L_ and BCLw [9], [11]. ABT-263 does not inhibit the activity of the other three BCL2-like proteins, BCLb, BFL1 and MCL1, and as a consequence expression of any of these three proteins can potentially lead to resistance to the drug [12], [13], [14]. Additional mechanisms have also been described that can cause cancer cells to become insensitive to ABT-263 treatment, such as loss of the pro-apoptotic BCL2 proteins, BAK or BIM [14], [15]. In addition, patients that receive ABT-263 display thrombocytopenia caused by the ability of ABT-263 to block the function of BCLxl [11], [16].

Like the family of BCL2-like proteins, sphingolipids are also known to regulate apoptosis [5]. Cellular levels of ceramide, a central molecule in sphingolipid metabolism, are elevated following treatment of cells with cytotoxic stimuli and inhibiting its generation blocks or delays cell death [17]. Ceramide generation occurs upstream of the execution phase of apoptosis and data suggest that ceramides plays an important role in MOMP [17], [18]. Ceramide generation has been shown to be regulated by BCL2-like proteins. For example, overexpression of anti-apoptotic BCL2-like proteins BCL2 and BCLx_L_ have been shown to inhibit ceramide generation and/or its ability to induce MOMP and apoptosis [19], [20], [21], [22], [23], . In addition, our previously published data indicate that the pro-apoptotic BCL2-like protein BAK is required for ceramide synthase-mediated long-chain ceramide generation during apoptosis [17]. Once generated ceramide can be metabolized via several pathways, resulting in the production of pro-proliferative members of the sphingolipid family. For example, ceramide glycosylation results in the production of glucosylceramide, a sphingolipid that promotes cellular proliferation. Likewise, ceramide can be broken down by the action of ceramidases to form sphingosine, which is utilized by sphingosine kinases to form sphingosine-1-phosphate, (S1P) another sphingolipid with anti-apoptotic and pro-proliferative roles. It is thought that the alteration in the balance between pro-apoptotic and pro-proliferative bioactive sphingolipids affects a cell’s ability to undergo apoptosis [5]. For example, elevated levels of glucosylceramide and the activity of glucosylceramide synthase, the enzyme responsible for its synthesis, occur in numerous cancers and inhibitors of glucosylceramide synthesis sensitize cancer cells to cytotoxic stimuli [26], [27], [28], [29]. Data also suggest that degradation of sphingosine kinase 1 occurs during apoptosis and leads to ceramide accumulation [30]. In addition, overexpression of sphingosine kinase 1 inhibits apoptosis [31]. Likewise, ceramide metabolism to sphingomyelin is elevated in particular types of cancers and thus sphingomyelin synthase is currently being explored as a potential target for cancer therapies [27]. Thus, it is not surprising that multiple drugs that regulate sphingolipid metabolism are currently being tested in preclinical and clinical for the treatment of human cancers.

In an effort to identify drugs that can sensitize cells to inhibition of anti-apoptotic BCL2-like proteins, we screened a library of compounds in the presence or absence of the first generation BCL2 inhibitor, ABT-737. The screen found that D,L-threo-1-phenyl-2-decanoylamino-3-morpholino-1-propanol (PDMP) hydrochloride, an inhibitor of glucosylceramide synthase (GCS), was capable of synergistically killing human leukemia cell lines when combined with ABT-737. We reasoned that drugs that are known to alter the balance between pro-apoptotic and pro-proliferative bioactive lipids may potentially sensitize cells that are otherwise resistant to inhibition of only BCL2-like proteins. Thus, we performed follow up validation experiments and the data demonstrate that co-treatment of cells with PDMP and the clinical BCL2 inhibitor, ABT-263, caused a significant elevation of ceramide and sphingosine when compared to either treatment alone. Importantly, an alternative inhibitor of GCS, AMP-deoxynijimycin (AMP), did not increase the levels of ceramide or sphingosine and was not capable of sensitizing cells to ABT-263 induced apoptosis. As further support that elevated ceramide and sphingosine are likely important in increasing ABT-263 induced apoptosis, we demonstrate that the combining ABT-263 with the sphingosine kinase inhibitor SKi-II also significantly increases ceramide and sphingosine levels. We propose a novel mechanism by which the combination of drugs that elevate ceramides by inhibiting their metabolism to pro-proliferative sphingolipids can be combined with inhibitors of BCL2-like proteins to eradicate human cancer cells.

## Materials and Methods

### Cell Culture

Human leukemia cell lines, U937, K562, HL60 and RPMI-8226 were obtained from ATCC (Manassas, VA, USA) and cultured in RMPI media containing 10% FBS, 1% L-glutamine and 1% Penicillin/Streptomycin. Cells were maintained according to the manufacturer’s protocol and were not cultured for more than thirty doublings. Cells were routinely assessed for mycoplasma using ‘MycoSensor PCR assay kit’ cat #302108 from Agilent Technologies (Santa Clara, CA, USA) according to manufacturer’s protocol. Both cell culture supernatant and cell lysates were tested. In addition, cells were routinely examined for morphological characteristics and were tested for consistent IC50 to ABT-263, since each line has a distinguishable level of sensitivity to this drug.

### LOPAC1280 Screen

Assays were conducted in sterile, tissue culture treated 1536-well white solid bottom tissue plates (cat#789173-F, Greiner Bio-One, Monroe, NC, USA). A total of 500 cells (RPMI-8226, HL-60 or U937) per well in 5 µL of phenol red free RPMI containing 5% FBS were seeded using a Multidrop Combi Reagent dispenser and a small pin cassette (Thermo Scientific, Fisher Scientific, Fair Lawn, NJ, USA). The appropriate amount of ABT-737 (from Chemietek, cat#CT-A737) or DMSO vehicle was added to each suspension of cells as it was plated. Immediately after dispensing the cells, 23 nL of compound solution from the LOPAC1280 (Sigma) in DMSO was transferred using a Kalypsys pintool. The plates were then covered with stainless steel Kalypsys lids and placed into incubator at 37°C, with 5% CO_2_ and 95% relative humidity. The plates were incubated for 48 hours and 3 µL of CellTiter-Glo® assay from Promega (Madison, WI, USA) was added using a BioRAPTR® (Beckton Coulter, Brea, CA, USA). Plates were incubated for 30 minutes at room temperature, spun at 1000 rpms and relative luciferase units (RLU) were quantified using a ViewLux (PerkinElmer, Waltham, MA). Spotfire DecisionSite for Lead Discovery was used to generate 3D scatter plots to visualize hits from the LOPAC library (50 µM) that were effective at reducing cell numbers in the presence of IC30 of ABT-737. Values are from each cell line are derived from DMSO normalized percent activity of Cell Titer Glo reagent where higher percentages represent more viable cells. The X-axis contains the U937 data, the Y-axis the RMPI8826 data and the Z-axis the HL60 data. The red arrow is highlighting the marked compound in Spotfire corresponding to NCGC00162323-02 or DL-Threo-PDMP.

### Relative Cell Viability Assays

Exponentially growing human leukemia cells were seeded in 96-well dishes (5,000 U937 cells or 7,500 K562 cells per well) and immediately treated with the indicated drug concentrations in a total volume of 100 µl per well. All treatments were done in triplicate. DL, PDMP (cat#10005276), AMP-DNM (cat#10010332) and SKi-II (cat#10009222) were obtained from Cayman Chemical (Ann Arbor, MI, USA) and ABT-263 (cat#CT-A263) was obtained from Chemietek (Indianapolis, IN, USA). Cells were incubated for 48 hours and 10 µl of Alamar Blue reagent from Invitrogen cat#DAL1100 (Grand Island, NY, USA) was added to each well. Plates were then incubated and the fluorescence of Alamar Blue reduction was determined on a SPECTRAmax Gemini EM plate reader every hour until untreated wells were mid-linear, approximately 4,000 arbitrary units (540 nm excitation, 594 nm emission). Wells containing only 100 µl of complete RPMI media plus 10 µl of Alamar Blue were averaged and subtracted from all experimental readings. Drug treatment regimens were then normalized to either vehicle treated cells or to wells containing only the IC30 of the indicated drug. Each graph shown is a representative experiment of at least three biological replicates. Isobologram analysis was performed for co-treatment experiments using the CalcuSyn software (Biosoft, Cambridge, UK).

### Apoptosis Assays

Cleaved CASP3 western blots - 5×10^6^ leukemia cells were seeded in each well of a 6-well dish in 4 ml total complete RPMI and treated with the indicated drug(s) or appropriate vehicle. At the indicated times, cells were harvested and lysed in an appropriate volume of NP-40 lysis buffer (1% NP-40, 10% glycerol, 137 mM NaCl, 20 mM Tris pH 8.0). Protein concentrations were determined using BCA protein assay reagent from Thermo Scientific (Rockford, IL, USA) cat# 23225. 30 µg of total protein was used for standard western blot procedure. Anti-cleaved CASP3 from Cell Signaling (Beverly, MA, USA) cat#9664 was used for western blotting at a dilution of 1∶2000, or anti-GAPDH from Santa Cruz Biotechnology (Santa Cruz, CA, USA) cat#sc-47724 was used at a dilution of 1∶2000. Chemiluminescent detection was performed using SuperSignal WestFemto from Thermo Scientific (Rockford, IL, USA) according to manufacturer’s protocol.

AnnexinV/7AAD flow cytometry - 1×10^6^ leukemia cells were seeded in each well of a 12-well dish in 2 ml total complete RPMI and treated with the indicated drug(s) or appropriate vehicle. At the indicated times, cells were harvested and resuspended in 500 µl of 1× AnnexinV binding buffer (10 mM Hepes, 140 mM NaCl, 25 mM CaCl_2_). 5 µl of APC conjugated anti-AnnexinV, cat#550474, and 5 µl of 7-AAD, cat#559925 (Becton-Dickinson, Franklin Lakes, NJ, USA), were added to the cells and incubated for 15 minutes at 4 degrees Celsius. Flow cytometry was performed using standard procedures on a 5-color FacScan from Becton-Dickinson (Franklin Lakes, NJ, USA).

### Sphingolipidomic Mass Spectrometry

5×10^6^ leukemia cells were seeded in each well of a 6-well dish in 4 ml total complete RPMI and treated with the indicated drug(s) or appropriate vehicle in biological triplicates. At the indicated times, cells were harvested, washed two times in 5 mL of ice cold 1× Phosphate buffered saline and then cell pellets were snap frozen in liquid nitrogen. Quantification of sphingolipids species was performed by the Lipidomics Core Facility at the Medical University of South Carolina (MUSC) on a Thermo Finnigan TSQ 7000, triple-stage quadrupole mass spectrometer operating in a Multiple Reaction Monitoring (MRM) positive ionization mode as described [32]. Data were normalized to total lipid phosphate.

## Results

### A Screen Identified PDMP as a Drug that Synergizes with ABT-737 to Inhibit Growth of Human Leukemia Cells

**Figure 1:**
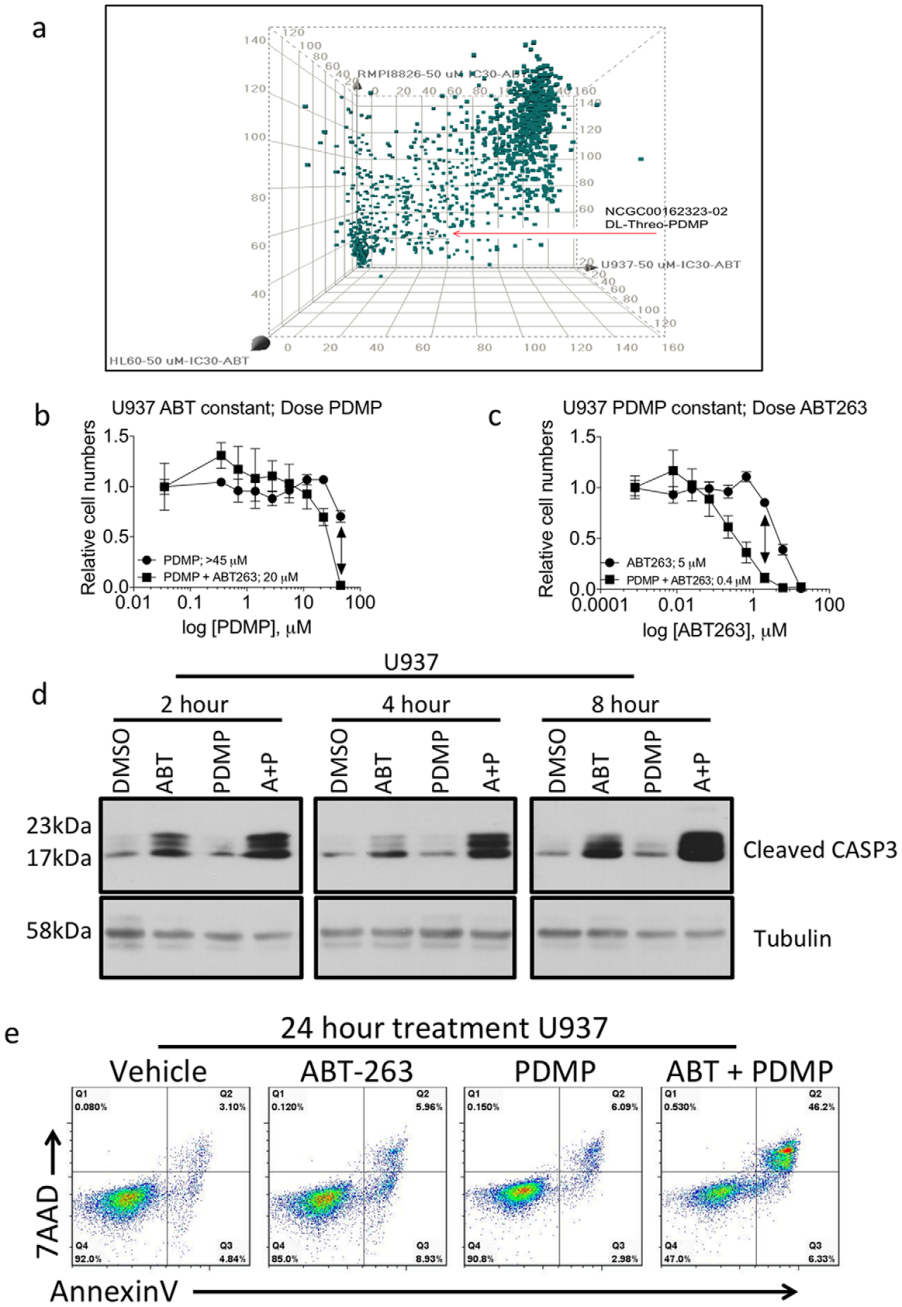
A screen identifies PDMP as a drug that can synergistically inhibit the growth of human leukemia cells when combined with ABT-263. (**A**) Three human leukemia cell lines were screened against the LOPAC1280 compound library with and without the IC30 of ABT-737. A synergy score was calculated for each compound in the library and the scores of each compound are plotted as a component of each cell line. A lower score indicates a higher level of synergy. U937 cells are plotted on the x-axis, RPMI8226 cells are plotted on the y-axis and HL60 cells are plotted on the z-axis. The point on the graph representing PDMP is indicated. (**B**) Dose response curves of U937 cells were determined by treating the cells with either increasing doses of PDMP (350 nM to 45 µM) plus vehicle or increasing doses of PDMP (350 nM to 45 µM) and a constant dose of ABT-263 (2 µM). Inset values are the calculated IC50 from each curve. (**C**) Dose response curves of U937 cells were determined by treating the cells with either increasing doses of ABT-263 (8 nM to 18 µM) plus vehicle or increasing doses of ABT-263 (8 nM to 18 µM) and a constant dose of PDMP (45 µM). Arrows in (**B**) and (**C**) represent the equivalent doses of the respective drugs (2 µM ABT-263, 45 µM PDMP) and isobologram analysis indicated that the combination of the two drugs was synergistic with CI = <0.1. Inset values are the calculated IC50 from each curve. (**D**) Cells were treated with ABT-263 (2 µM), PDMP (45 µM) or the combination of drugs for 2, 4, or 8 hours and western blots for cleaved CASP3 were performed. (**E**) Cells were treated with either ABT-263 (2 µM), PDMP (45 µM) or the combination of drugs and 24 hours post treatment cells were stained with anti-AnnexinV antibody and 7AAD to determine the percent of cells undergoing apoptosis.

To identify combinations of compounds that synergistically inhibit the growth of human leukemia cells we performed a small molecule screen by combining the BCL2 inhibitor, ABT-737 with 1280 drugs in the library of pharmacologically active compounds (LOPAC1280). The library was screened at multiple concentrations (45 µM, 9 µM and 1.8 µM) with and without the IC30 or IC70 of ABT-737. We used three human leukemia cell lines of different lineage, RPMI8226, U937 and HL60 cells, to increase the potential of discovering synergistic compounds capable of broadly inhibiting leukemia cell growth. Cells were cultured for 48 hours in the presence of drugs and then relative cell numbers were determined by cell-titre glow assay. Synergy scores were calculated for each drug in each cell line (**Fig. 1A**). Of interest to our lab was that a known modulator of ceramide metabolism D,L-threo-1-phenyl-2-decanoylamino-3-morpholino-1-propanol (PDMP) hydrochloride that ranked among the highest synergy scores across all three cell lines. To validate the results from the screen, we treated U937 cells with two different regimens of PDMP and the clinically utilized BCL2 inhibitor, ABT-263. First, cells were treated with increasing doses of PDMP alone (350 nM to 45 µM) or increasing doses of PDMP with the pre-determined IC30 of ABT-263 (2 µM) (**Fig. 1B**). Second, we treated cells with increasing concentrations of ABT-263 (8 nM to 18 µM) alone or increasing amounts of ABT-263 combined with 45 µM of PDMP (**Fig. 1C**). We observed a significant decrease in the relative number of cells when the two drugs were combined, compared to the single treatments. In fact, isobologram analysis demonstrated that the combination idex (CI) of the points corresponding to 2 µM of ABT-263 and 45 µM of PDMP was less than 0.1.

Since we observed a dramatic reduction in the number of viable cells following co-treatment with ABT-263 and PDMP we wanted to explore the mechanism by which the relative numbers of cells were reduced. To this end, we treated the U937 cells with each drug individually or in combination and looked at 2, 4 and 8 hours post treatment for increased cleaved Caspase3 (cCASP3), which is the active form of the apoptotic executioner (**Fig. 1D**). As early as 2 hours post co-treatment, there was an increase in the amount of activated cCASP3 and by 8 hours the increase in cCASP3 of co-treated cells was dramatic compared to either single drug treatment alone. To further demonstrate that the treatments were leading to apoptotic cell death, we performed single or co-treatments followed by flow cytometry with anti-annexinV and 7AAD. After 24 hours of treatment, there was a significant increase in the percentage of cells undergoing apoptosis in the co-treatment when compared to the cells receiving single treatments (**Fig. 1E**).

**Figure 2:**
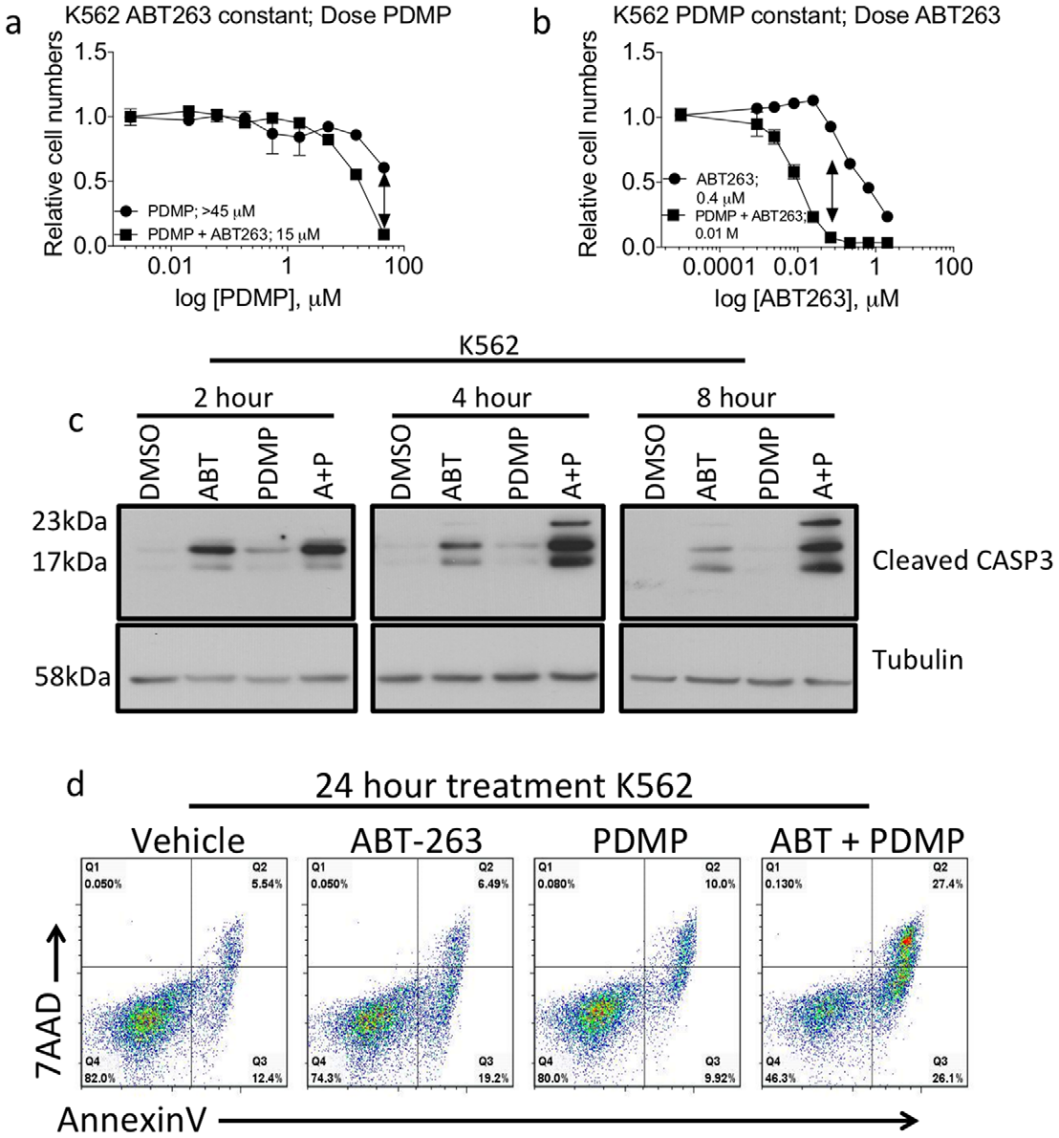
Validation of synergy in K562 cells, a line not used in the screen. (**A**) Dose response curves of K562 cells were determined by treating the cells with either increasing doses of PDMP (350 nM to 45 µM) plus vehicle or increasing doses of PDMP (350 nM to 45 µM) and a constant dose of ABT-263 (60 nM). Inset values are the calculated IC50 from each curve. (**B**) Dose response curves of K562 cells were determined by treating the cells with either increasing doses of ABT-263 (2.2 nM to 5 uM) plus vehicle or increasing doses of ABT-263 (2.2 nM to 5 µM) and a constant dose of PDMP (45 µM). Arrows in (A) and (B) represent equivalent doses of the respective drugs (60 nM ABT-263, 45 µM PDMP) and isobologram analysis indicated that the combination of the two drugs was synergistic with CI = <0.1. Inset values are the calculated IC50 from each curve. (**C**) Cells were treated with ABT-263 (60 nM), PDMP (45 µM) or the combination of drugs for 2, 4, or 8 hours and western blots for cleaved CASP3 were performed. (**D**) Cells were treated with either ABT-263 (60 nM), PDMP (45 µM) or the combination of drugs and 24 hours post treatment cells were stained with anti-AnnexinV antibody and 7AAD to determine the number of cells that were undergoing apoptosis or were already dead.

To further validate the potency of combining ABT-263 and PDMP, we utilized a human leukemia cell line not included in the original drug screen. K562 cells were treated with increasing doses of PDMP alone (350 nM to 45 µM) or increasing doses of PDMP with the pre-determined IC30 of ABT-263 (60 nM) (**Fig. 2A**). Next, we treated K562 cells with increasing concentrations of ABT-263 (2.2 nM to 5 µM) alone or increasing doses of ABT-263 combined with 45 µM of PDMP (**Fig. 2B**). We observed a significant decrease in the relative number of cells when the two drugs were combined, compared to the single treatments. As before, isobologram analysis demonstrated that the combination idex (CI) of the points corresponding to 60 nM of ABT-263 and 45 µM of PDMP was less than 0.1.

As with the experiments performed in U937 cells, we wanted to examine the mechanism by which co-treatment of K562 with ABT-263 and PDMP was leading to such a dramatic decrease in the relative cell numbers. We observed rapid and synergistic activation of apoptosis by both western blot for cCASP3 and by anti-AnnexinV/7AAD flow cytometery (**Fig. 2C and 2D**). These data indicate that ABT-263 and PDMP synergistically induce apoptosis of human leukemia cells and suggest that co-treatment with ABT-263 and PDMP is a potent combinatorial therapeutic option.

### AMP-Deoxynojirimycin does not Synergize with ABT-263 to Kill Human Leukemia Cells

**Figure 3:**
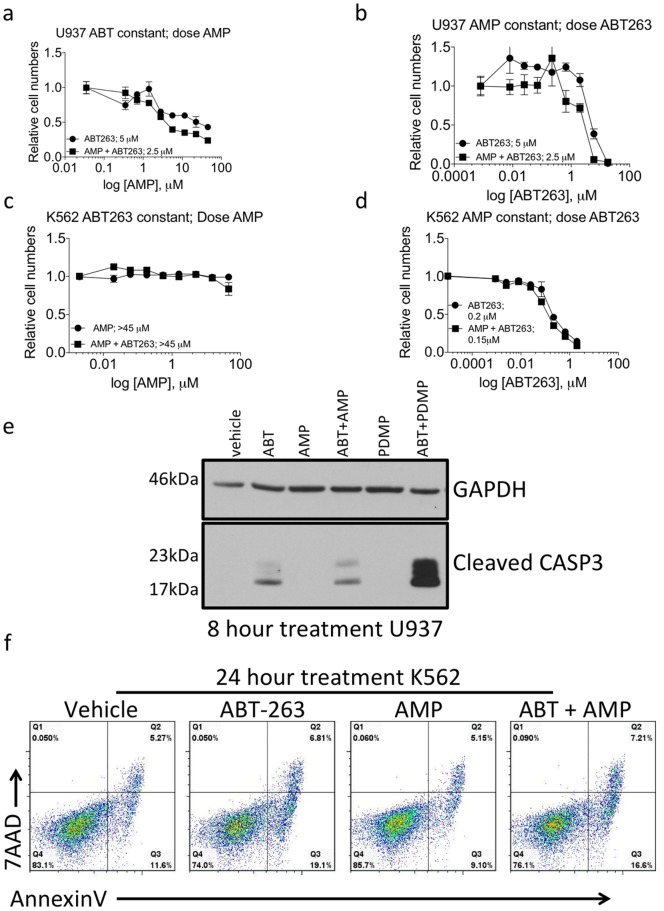
Treatment of cells with AMP-deoxynojirimycin does not synergize with ABT-263. (**A**) and (**C**) Dose response curves of U937 cells and K562 were determined by treating the cells with either increasing doses of AMP (350 nM to 45 µM) plus vehicle or increasing doses of AMP (350 nM to 45 µM) and a constant dose of ABT-263 (2 µM or 60 nM, respectively). (**B**) and (**D**) Dose response curves of U937 cells and K562 cells were determined by treating the cells with either increasing doses of ABT-263 (8 nM to 18 µM for U937, 2.2 nM to 5 µM for K562) plus vehicle or increasing doses of ABT-263 (8 nM to 18 µM for U937, 2.2 nM to 5 µM for K562) and a constant dose of AMP (45 µM). Inset values are the calculated IC50 from each curve. (**E**) U937 cells were treated with ABT-263 (2 µM), PDMP (45 µM) or the combination of drugs for 8 hours and western blots for cleaved CASP3 were performed. (**F**) Cells were treated with either ABT-263 (60 nM), PDMP (45 µM) or the combination of drugs and 24 hours post treatment cells were stained with anti-AnnexinV antibody and 7AAD to determine the number of cells that were undergoing apoptosis or were already dead.

PDMP is a glucosylceramide synthase (GCS) inhibitor. Thus, we wanted to determine if another inhibitor of GCS, AMP-Deoxynojirimycin (AMP-DNM) would also synergize with ABT-263 to eradicate human leukemia cells. U937 and K562 cells were treated as before by either increasing the dose of AMP-DNM and holding ABT-263 constant, or *vice versa*. In contrast to PDMP, we did not observe any increase in the potency of the co-treatments compared to either single treatment alone (**Fig. 3A, 3B, 3C, 3D**). In addition, ABT-263 and AMP-DNM co-treatment did not increase apoptosis in either U937 or K562 (**Fig. 3E and 3F**). These data indicate that there must be alternative signals that are activated in cells following treatment with PDMP or AMP-DNM that dictate the ability of the two drugs to differentially cooperate with inhibition of BCL2-like proteins in the killing of human leukemia cells.

### PDMP Plus ABT-263 Cause Increased Ceramide Accumulation in Cells

**Figure 4:**
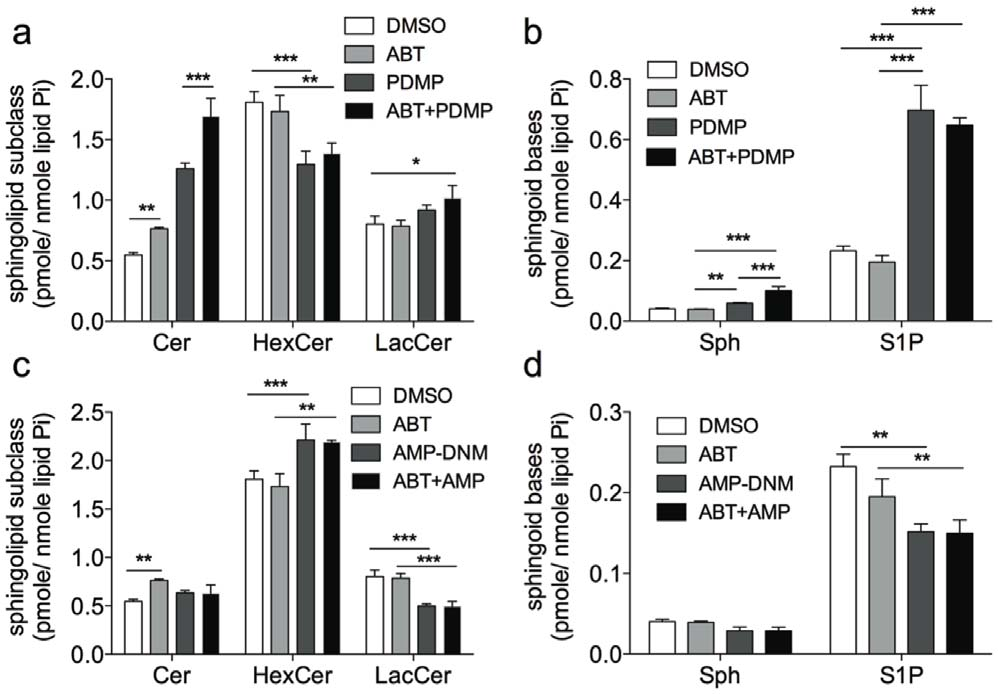
Treatment of U937 cells with PDMP, but not AMP-DNM causes accumuliation of ceramide and sphingosine and a decrease in glucosylceramide. (**A**) U937 cells were treated with ABT-263 (2 µM), PDMP (45 µM) or the combination of both drugs from two hours. Cells were harvested, lipids were extracted and the amounts of ceramide (Cer), hexosyceramine (HexCer) and lactosylceramide (LacCer) were quantitated. (**B**) U937 cells were treated with ABT-263 (2 µM), PDMP (45 µM) or the combination of both drugs from two hours. Cells were harvested, lipids were extracted and the amounts of sphingosine (Sph) and spingosine-1 phosphate (S1P) were quantitated. (**C**) U937 cells were treated with ABT-263 (2 µM), AMP-DNM (45 µM) or the combination of both drugs from two hours. Cells were harvested, lipids were extracted and the amounts of ceramide (Cer), hexosyceramine (HexCer) and lactosylceramide (LacCer) were quantitated. (**D**) U937 cells were treated with ABT-263 (2 µM), AMP-DNM (45 µM) or the combination of both drugs from two hours. Cells were harvested, lipids were extracted and the amounts of sphingosine (Sph) and spingosine-1 phosphate (S1P) were quantitated.

PDMP is known to elevate cellular ceramide and decrease glucosylceramides via inhibition of glucosylceramide synthase [33]. Alternatively, AMP-DNM was designed to avoid an accumulation of ceramides via inhibition of multiple enzymes in the glycosphingolipid pathway [34]. We therefore reasoned that ceramide accumulation and/or the combined effects of elevated ceramides with decreased glucosylceramides might play a role in the synergism of PDMP with ABT-263 in the induction of human leukemia cell apoptosis. To this end, we quantified cellular ceramides, sphingoid bases, monohexosylceramides (the combined quantification of glucosylceramides and galactosylceramides), and lactosylceramides via HPLC-MS/MS following treatment of U937 cells with ABT-263, PDMP, AMP-DNM or the combination of ABT-263 with either PDMP or AMP-DNM. The majority of cells were already undergoing later stages of apoptosis by 8 hours following co-treatment with ABT-263 and PDMP. Thus, sphingolipds were measured following 2 and 8 hours of treatment in order to increase the likelihood that we were identifying changes that were causal to apoptosis, rather than a consequence of apoptosis. Treatment of U937 cells with ABT-263 alone lead to a significant increase in the total cellular ceramide, while not significantly affecting any other measured lipids (**Fig. 4A and 4B**). PDMP alone was sufficient to elevate ceramide and sphingosine, while decreasing levels of total hexosylceramides (**Fig. 4A and 4B**). PDMP treatment resulted in elevated levels of S1P; this is most likely due to the PDMP-induced ceramide accumulation, which is then metabolized to S1P through sphingosine. On the contrary, AMP-DNM treatment alone did not increase total cellular ceramides or sphingosine and actually increased hexosylceramides (**Fig. 4C and 4D**). Interestingly, when U937 cells were co-treated with both ABT-263 and PDMP for 2 hours there was a significant increase in ceramide and sphingosine beyond the elevation caused by either single drug (**Fig. 4A and 4B**). In stark contrast, co-treatment with AMP-DNM and ABT-263 did not induce ceramide or sphingosine accumulation (**Fig. 4C and 4D**) and actually elevated hexosylceramides (**Fig. 4C**). These results suggest that the ability of PDMP to synergize with ABT-263 in killing leukemia cells is dependent on its ability to increase pro-apoptotic sphingolipids (ceramide and sphingosine) and reduce pro-proliferative sphingolipids (glucosylceramides).

### Inhibition of Sphingosine Kinase also Synergizes with ABT-263 to Kill Leukemia Cells

**Figure 5:**
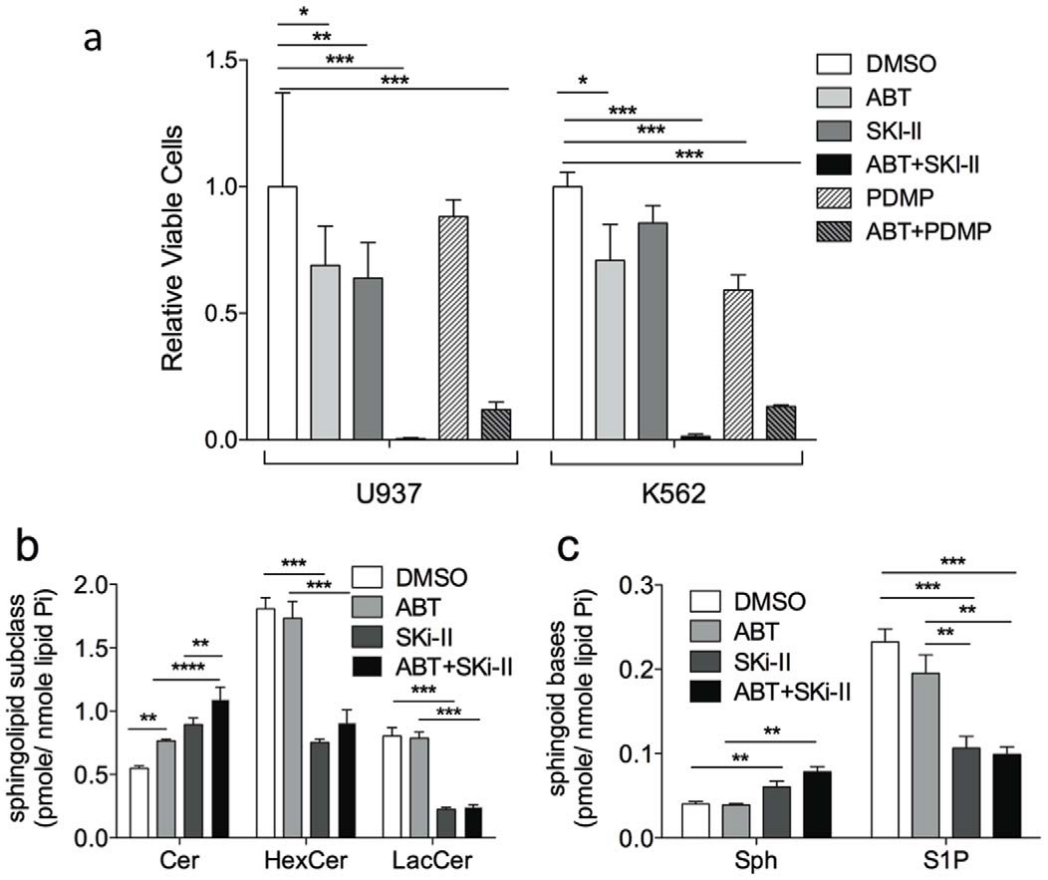
Inhibition of sphingosine kinase synergizes with ABT-263 to kill leukemia cells by causing accumulation of ceramide and sphingosine while decreasing glucosylceramide and S1P. (A) U937 and K562 cells were treated with single doses of ABT-263 (2 µM U937, 60 nM K562), PDMP (45 µM), SKi-II (5 µM) or combinations of the indicated concentrations of ABT-263 and PDMP or ABT-263 and SKi-II and then alamar blue was used to determine the relative cell numbers after 48 hours of treatment. (**B**) U937 cells were treated with ABT-263 (2 µM), SKi-II (5 µM) or the combination of both drugs from two hours. Cells were harvested, lipids were extracted and the amounts of ceramide (Cer), hexosyceramine (HexCer) and lactosylceramide (LacCer) were quantitated. (**C**) U937 cells were treated with ABT-263 (2 µM), SKi-II (5 µM) or the combination of both drugs for two hours. Cells were harvested, lipids were extracted and the amounts of sphingosine (Sph) and spingosine-1 phosphate (S1P) were quantitated.

Inhibition of glucosylceramide synthase is only one of many mechanisms that would lead to accumulation of cellular ceramide. Sphingosine kinase (SK) generates sphingosine-1-phosphate (S1P) from sphingosine, an immediate metabolite of ceramide. Therefore, to determine if inhibition of SK would also increase levels of both sphingosine and ceramide and sensitize cells to ABT-263 we treated U937 and K562 cells with the SK inhibitor, SKi-II. Cells were treated with ABT-263, PDMP, SKi-II, ABT-263 and PDMP or ABT-263 and SKi-II and assessed by Alamar blue for relative cell numbers 48 hours post treatment (**Fig. 5A**). As before, ABT-263 and PDMP was a potent combination for reducing the total number of viable cells., Treatment of cells with ABT-263 and SKi-II was an even more potent combination, leading to an almost complete eradication of cells during the treatment course (**Fig. 5A**). As above, sphingolipids were quantified in U937 cells 2 hours following treatment with ABT-263, SKi-II or the combination of inhibitors. Treatment of cells with SKi-II alone increased ceramide and sphingosine while decreasing the levels of both hexosylceramides and S1P (**Fig. 5B and 5C**). As with the combination of PDMP and ABT-263, combining SKi-II with ABT-263 led to a significant increase in both ceramide and sphingosine, when compared to either drug alone (**Fig. 5B and 5C**). From the combination of these experiments, a role for S1P or lactosylceramide in the sensitization of cells to ABT-263 can be ruled out. Data are consistent with a role for elevated ceramide and sphingosine and decreased hexosylceramides in sensitizing human leukemia cells to lower doses of ABT-263.

### CCRF-CEM Cells are not Synergistically Inhibited by the Combination of SKi-II and ABT-263

**Figure 6:**
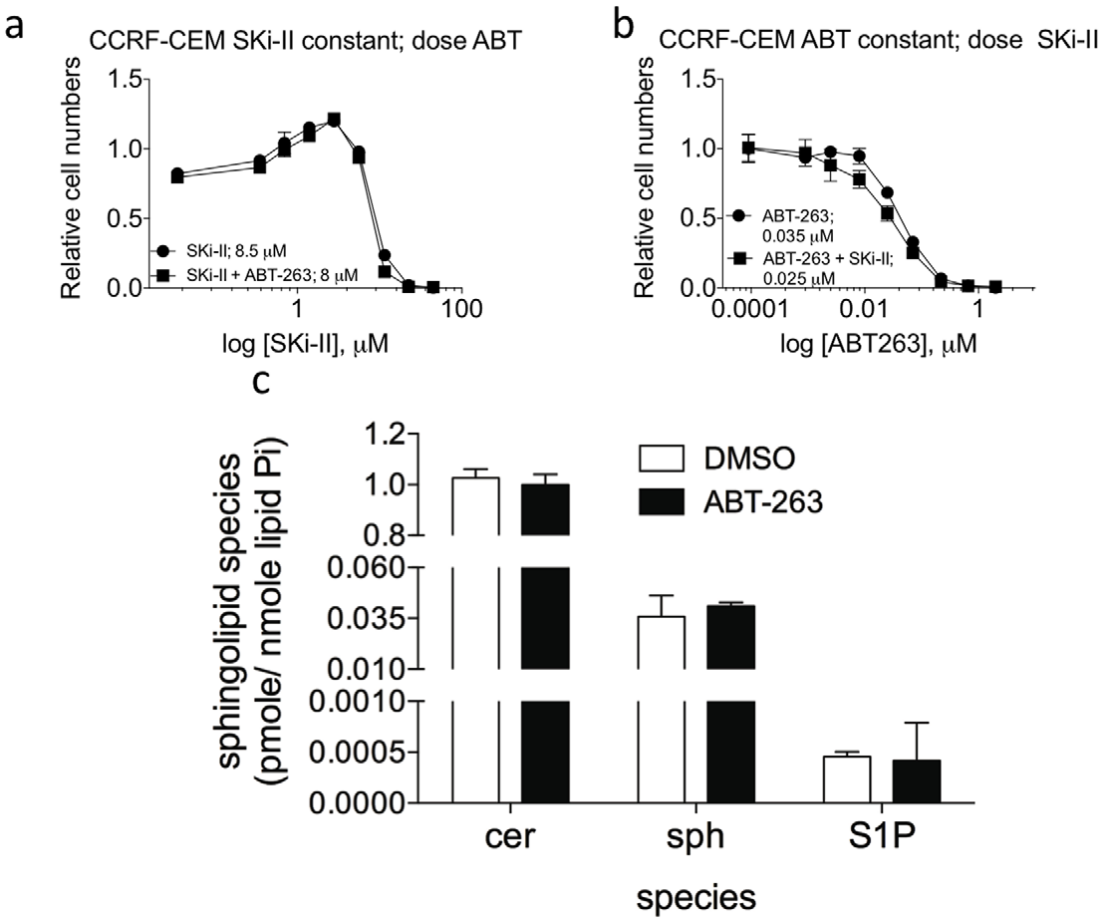
Inhibition of sphingosine kinase does not synergize with ABT-263 in killing CCRF-CEM cells. (**A**) Dose response curves of CCRF-CEM cells were determined by treating the cells with either increasing doses of SKi-II (350 nM to 45 µM) plus vehicle or increasing doses of PDMP (350 nM to 45 µM) and a constant dose of ABT-263 (20 nM). Inset values are the calculated IC50 from each curve. (**B**) Dose response curves of CCRF-CEM cells were determined by treating the cells with either increasing doses of ABT-263 (0.9 nM to 2 µM) plus vehicle or increasing doses of ABT-263 (0.9 nM to 2 µM) and a constant dose of SKi-II (2 µM). Inset values are the calculated IC50 from each curve. (**C**) CCRF-CEM cells were treated with the IC30 of ABT-263 (20 nM) or vehicle (DMSO) for eight hours. Cells were harvested, lipids were extracted and the amounts of ceramide, sphingosine (Sph) and spingosine-1 phosphate (S1P) were quantitated.

One important question that remains to be answered is whether co-treatment with inhibitors of ceramide metabolism, such as SKi-II, and ABT-263 will be toxic to all cells. To this end, we have found that treatment of the T-cell lymphoblastic leukemia cell line CCRF-CEM with SKi-II and ABT-263 does not synergistically kill the cells (**Fig. 6A and 6B**). CCRF-CEM cells were treated with increasing doses of SKi-II alone (350 nM to 45 µM) or increasing doses of SKi-II with the pre-determined IC30 of ABT-263 (20 nM) (**Fig. 6A**). Next, we treated CCRF-CEM cells with increasing concentrations of ABT-263 (0.9 nM to 2 µM) alone or increasing doses of ABT-263 combined with 2 µM of SKi-II (**Fig. 6B**). We observed no significant decrease in the relative number of cells when the two drugs were combined, compared to the single treatments indicating that SK inhibition does not further sensitize CCRF-CEM cells to ABT-263 induced apoptosis.

The finding that SKi-II and ABT-263 could not synergize to kill CCRF-CEM cells led us to examine the potential mechanism. From the compilation of our data we hypothesized that ABT-263 induced ceramide accumulation is critical for the ability of drugs that block ceramide metabolism to synergize with ABT-263 in killing cells. Therefore, we treated CCRF-CEM cells with ABT-263 and examined the levels of total ceramide, sphingosine and S1P. In agreements with the other cell lines tested, ABT-263 did not induce sphingosine or S1P in CCRF-CEM cells. However, in stark contrast to cell lines that are sensitized to the combination of either PDMP or SKi-II, treatment of CCRF-CEM cells with ABT-263 did not lead to any increase in total ceramides (**Fig. 6C**). These data support the notion that the ceramide accumulation induced by ABT-263 is critical for the ability of ceramide metabolism inhibition to synergize in the killing of human leukemia cells.

## Discussion

The two major hurdles to the development of targeted cancer-specific therapies are primary (or acquired) resistance and a lack of true cancer specificity that manifests as detrimental patient side-effects. One way to combat both of these pitfalls is to identify combinations of therapies that work synergistically to kill the cancer cells. These combinations would decrease the likelihood that cancer cells would be able to develop resistance. In addition, synergistic drug combinations will kill the cancer cells at much lower doses than would be required if the drugs were administered independently, reducing the likelihood of patient side-effects. Herein, we demonstrate for the first time that the combination of drugs that inhibit the BCL2-like family of proteins with drugs that increase the ratio of pro-apoptotic to pro-proliferative bioactive sphingolipids are potential potent and synergistic cancer therapies.

Our initial screen was designed to identify drugs that could be combined with inhibition of BCL2-like proteins to synergistically kill human leukemia cells. This screen was performed on three different leukemia types with the goal of finding combinations that would be effective against multiple leukemia types rather than a single lineage of leukemia. To our surprise, we found many positive hits that scored as highly synergistic across all three cell lines. In addition, many drugs were identified as being synergistic in only one or two of the lines tested. These results are currently being validated.

**Figure 7:**
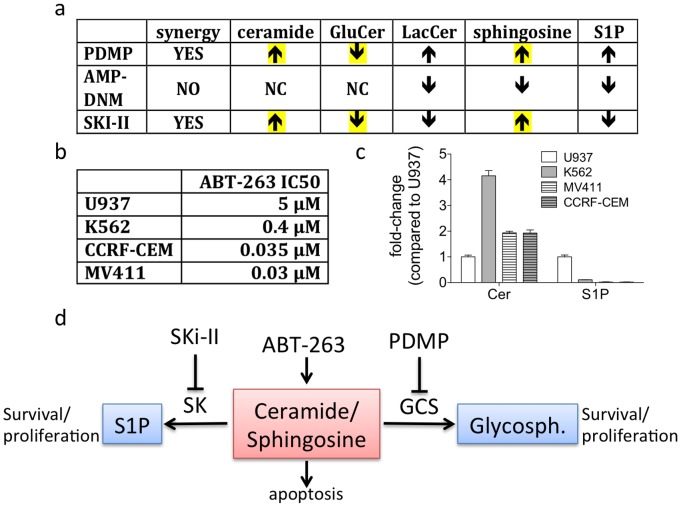
Increased ceramide and sphingosine are important for synergy with ABT-263. (**A**) Summary of the lipids that are altered following treatment of U937 cells with various inhibitors. (**B**) IC50 values of ABT-263 for individual human leukemia cell lines. Values were calculated in prism as the result of dose response curves determined by alamar blue assay 48 hours after ABT-263 treatment. (**C**) Total levels of basal ceramide (Cer) and sphingosine-1-phosphate (S1P) in four different cell lines as determined by HPLC-MS/MS. Data are normalized to the levels of lipids in U937. (**D**) Model depicting how different inhibitors affect sphingolipid metabolism.

The finding that PDMP had a positive synergy score in all three leukemia cell lines in the screen was validated independently in all three cell lines (**Fig. 1** **,** **Fig. 2** **, and not shown**). By combining the cell culture based drug treatments of ABT-263 and PDMP, with unbiased total cellular sphingolipidomic profiles, we have also determined the potential mechanism for how the combinations of drugs are able to synergize in killing human leukemia cells (**Fig. 7A**). This mechanism is based on data presented herein and our previous findings that activation of BAK is capable of leading to increased ceramide synthase (CerS) activity [17]. We propose a model for synergistic killing of human cancer cells that relies on the combined increase in production of ceramide, likely through ABT-263-induced BAK activation, with inhibition of ceramide metabolism to its pro-proliferative metabolites glucosylceramide or sphingosine-1-phosphate, by either inhibition of glucosyceramide synthase (GCS) or inhibiton of sphingosine kinase (**Fig. 7A**), respectively. In support of this model, a drug that was designed to inhibit GCS, but not lead to the accumulation of ceramide, AMP-DNM was not capable of sensitizing cells to treatment with ABT-263, suggesting that synergism with ABT-263 requires at least in part the elevation of ceramide.

To determine if there is any correlation between the basal levels of sphingolipids and sensitivity to ABT-263, we first examined the IC50 for each of the cell lines used herein, and one additional cell line, MV411 (**Fig. 7B**). Then by compiling the data throughout this work and displaying it as a fold-change compared to the most ABT-263-resistant cell line, U937, we saw a striking correlation. First, there is a inverse correlation between the IC50 for ABT-263 and the basal level of pro-apoptotic ceramide (**Fig. 7C**). Second, there was an direct correlation between the IC50 for ABT-263 and the basal levels of pro-proliferative S1P. These observations are consistent with the known roles for ceramide and S1P in regulating apoptosis and survival, respectively (**Fig. 7C**). Information gained from our initial screen and quantitative sphingolipidomic analysis demonstrate that multiple drugs known to affect the balance between pro-apoptotic and pro-proliferative sphingolipids can be combined with ABT-263 to eradicate human leukemia cells (**Fig. 7D**). Future studies will be aimed at obtaining and testing clinically used inhibitors of BCL2-like proteins and modulators of sphingolipid metabolism, as well as determining the precise mechanism by which these drugs can synergize to eliminate leukemia cells.

The ability of ABT-263 alone to induce the generation of ceramide fits with our previously published data indicating a role for BAK in ceramide metabolism [17]. BAK is a post-translational regulator of long-chain ceramide synthase (CerS) activity and is required for long-chain CerS activity both basally and in cells undergoing apoptosis [17]. ABT-263 was designed to bind to the hydrophobic pocket of anti-apoptotic BCL2 proteins and release bound pro-apoptotic BCL2 proteins such as BAK [9]. Indeed, treatment of cells with ABT-263 is sufficient to induce activation of BAK and induce apoptosis [12]. The fact that ABT-263 treatment can induce ceramide generation could be explained by increased availability of BAK to activated CerS. Overexpression of BCL2 and/or BCLx_L_ blocks ceramide generation following treatment of cells with particular cytotoxic stimuli, which could be due to their binding to BAK and decreasing the concentration of free BAK available to interact with CerS. Thus, ABT-263 treatment could induce apoptosis in part by induction of ceramide generation. Once generated, ceramides are known to be important for induction of MOMP. Ceramides have been shown to induce the formation of channels in membranes capable of allowing the passage of proteins [18], [35]. Importantly, ceramide channels are inhibited by anti-apoptotic BCL2 proteins such as BCLx_L_ [4], [36] and ABT-263 prevents BCLx_L_ inhibition of ceramide channels [4]. In addition, ceramide generation is upstream of, and has been reported to be important for, BAX activation in cells [37], [38]. Indeed, *in vitro* data in isolated mitochondria suggest that ceramide metabolites regulate MOMP induction via activation of BAX and BAK [39]. Recent reports indicate that ceramide and BAX synergize to form a novel channel with characteristics consistent with those required for MOMP induction [40]. Although several mechanisms have been proposed for ceramide-induction of MOMP during apoptosis, most, if not all, of the literature suggests that it is via an interaction between ceramides (or one of its metabolites) and BCL2 proteins. Thus, due to the intimate interplay between BCL2-like family members and bioactive sphingolipids in apoptotic regulation, drugs that target both ceramide metabolism and anti-apoptotic BCL2 proteins may be good candidates for synergistic cancer therapies.

The ability of PDMP and SK inhibitors (SKi) to synergize with cytotoxic stimuli in the killing of cancer cells has been demonstrated by many groups and in a multitude of cancer types [26], [41], [42], [43], [44]. In fact, the types of apoptotic stimuli range from generally cytotoxic treatments, such as ionizing irradiation and paclitaxel, to more targeted therapeutics like imatinib and nilotinib. The ability of PDMP and SKi to synergize with cytotoxic compounds is not a universal finding, however. One curious observation suggests that treatment of leukemia cells with PDMP actually inhibits apoptosis induced by the alkylating agent daunorubicin, which is in stark contrast to the ability of SKi to synergize with daunorubicin in killing leukemia cells [45], . Another curious finding is that the natural phenolic compound resveratrol is capable of inducing apoptosis of multiple human leukemia cell lines and both PDMP and SKi cooperate with resveratrol in apoptotic induction [42]. Resveratrol is generally thought to be cytoprotective and has been implicated as a compound that can reduce inflammation and lead to increased lifespan in model organisms. However, multiple groups have demonstrated a role for resveratrol in the inhibition of SK1 activity and expression [47], [48]. Whether or not the inhibition of SK1 is the major mechanism by which resveratrol is able to cause leukemic cell death is still under investigation. In fact, the exact molecular mechanism by which the combination of PDMP or SKi with cytotoxic therapies leads to increased cell death is not clear, but almost always correlates with the accumulation of pro-apoptotic sphingolipids, such as ceramide. Herein, we have demonstrated that drugs that lead to the accumulation of ceramide and sphingosine are effective combinatorial therapies when used with molecules that block the function of anti-apoptotic BCL2 proteins. These results are of direct clinical significance because a multitude of BCL2-family inhibitors are currently being tested in human patients for the treatment of both solid cancers and leukemias [11], [13], [16]. In addition, there are currently FDA approved drugs that inhibit various enzymes involved in the metabolism of ceramide, as well as novel inhibitors that are being tested in early phase clinical trials [49], [50]. Future studies will be designed with the intention of testing combinations of these FDA approved and clinical candidates for synergistic killing of human leukemia cells *in vitro* and *in vivo* with the hope of bringing novel combinations of effective and safe drugs to patients.
